# A novel compound heterozygous variant of *ECHS1* identified in a Japanese patient with Leigh syndrome

**DOI:** 10.1038/s41439-019-0050-1

**Published:** 2019-04-19

**Authors:** Shumpei Uchino, Aritoshi Iida, Atsushi Sato, Keiko Ishikawa, Masakazu Mimaki, Ichizo Nishino, Yu-ichi Goto

**Affiliations:** 1 Department of Mental Retardation and Birth Defect Research, National Institute of Neuroscience, National Center of Neurology and Psychiatry (NCNP), Tokyo, Japan; 2 Department of Pediatrics, Teikyo University School of Medicine, Tokyo, Japan; 3 Department of Pediatrics, Graduate School of Medicine, The University of Tokyo, Tokyo, Japan; 4 Medical Genome Center, NCNP, Tokyo, Japan; 5 Department of Neuromuscular Research, National Institute of Neuroscience, NCNP, Tokyo, Japan

**Keywords:** Genetics research, Disease genetics

## Abstract

Leigh syndrome (LS) is a heterogeneous neurodegenerative disorder caused by mitochondrial dysfunction. Certain LS cases have mutations in *ECHS1*, which encodes a short-chain enoyl-CoA hydratase involved in the metabolism of fatty acids and branched-chain amino acids in mitochondria. Using exome sequencing, we diagnosed a Japanese patient with LS and identified the patient as a compound heterozygote for a novel variant of *ECHS1*, consisting of NM_004092.4:c.23T>C (p.Leu8Pro) and NM_004092.4:c.176A>G (p.Asn59Ser).

Leigh syndrome (LS; MIM #256000), the most common phenotype of pediatric mitochondrial disease, is characterized by symmetrical central nervous system lesions, especially in the basal ganglia and brainstem, and increased lactate in the blood and cerebrospinal fluid. Patients present with progressive neurological symptoms, including psychomotor developmental delay or regression, hypotonia, and involuntary movements. LS is caused by mutations in either mitochondrial or nuclear genes. To date, more than 75 causative genes have been reported^1^.

*ECHS1* on chromosome 10q26.3 encodes a short-chain enoyl-coenzyme A hydratase that localizes in the mitochondrial matrix and catalyzes the hydration of enoyl-CoA in many metabolic pathways, including short-chain fatty acid β-oxidation and branched-chain amino acid catabolism. Recently, *ECHS1* has been reported as a novel causative gene for LS^2,3^. To date, a total of 33 mutations associated with LS have been reported (HGMD Professional 2018.3). Dysfunction of ECHS1 protein is suspected to cause brain pathology through the accumulation of toxic metabolites and the impairment of energy production.

In this paper, we report a Japanese patient with LS who is a compound heterozygote for the novel *ECHS1* variant: c.23T>C (p.Leu8Pro) and c.176A>G (p.Asn59Ser).

The patient is a female with nonconsanguineous Japanese parents. She was born at 40 weeks of gestation by normal vaginal delivery after an uneventful pregnancy, and her birth measurements were normal. An auditory screening test at 1 month revealed severe hearing impairment. The patient also showed poor response to visual stimuli. Her early motor development was normal, and she could control her head at 3 months and roll over at 6 months. At 10 months, however, she became hypotonic and less responsive to light and sound. She was referred to pediatric neurologists at the age of 1 year on account of developmental regression. On physical examination, she showed muscle hypotonia and involuntary movements such as dystonia and choreoathetosis, along with almost complete unresponsiveness to visual and auditory stimuli. The lactate levels in the patient’s blood and cerebrospinal fluid were elevated to 53.9 mg/dL (reference range: 4.0–19.2) and 53.9 mg/dL (reference range: 4.0–19.2), respectively. Metabolic screening, including plasma amino acid analysis, showed no abnormal findings. Brain magnetic resonance imaging showed bilateral T2 hyperintensity in the caudate nucleus, putamen, globus pallidus, and substantia nigra (Fig. 1a, b). Ophthalmological examination revealed optic nerve atrophy. The auditory brainstem response showed bilateral sensorineural deafness. The patient was diagnosed with LS. Treatments including thiamine and coenzyme Q10 were administered, but were found to be ineffective. Subsequently, the patient’s hypotonia and involuntary movements intensified, and she also developed epilepsy. A gastrostomy was performed due to dysphagia and gastroesophageal reflux. A skeletal muscle biopsy performed at 2 years showed no pathological findings suggesting mitochondrial diseases. At 11 years of age, the patient had severe psychomotor developmental delay, no head control and no eye contact, and her refractory involuntary movements persisted. She needed noninvasive positive-pressure ventilation for central and obstructive apnea. The clinical information and materials from the patient were obtained for diagnostic purposes with written informed consent. All materials and experiments in this study were approved by the Ethical Committee of the National Center of Neurology and Psychiatry.

**Fig. 1:**
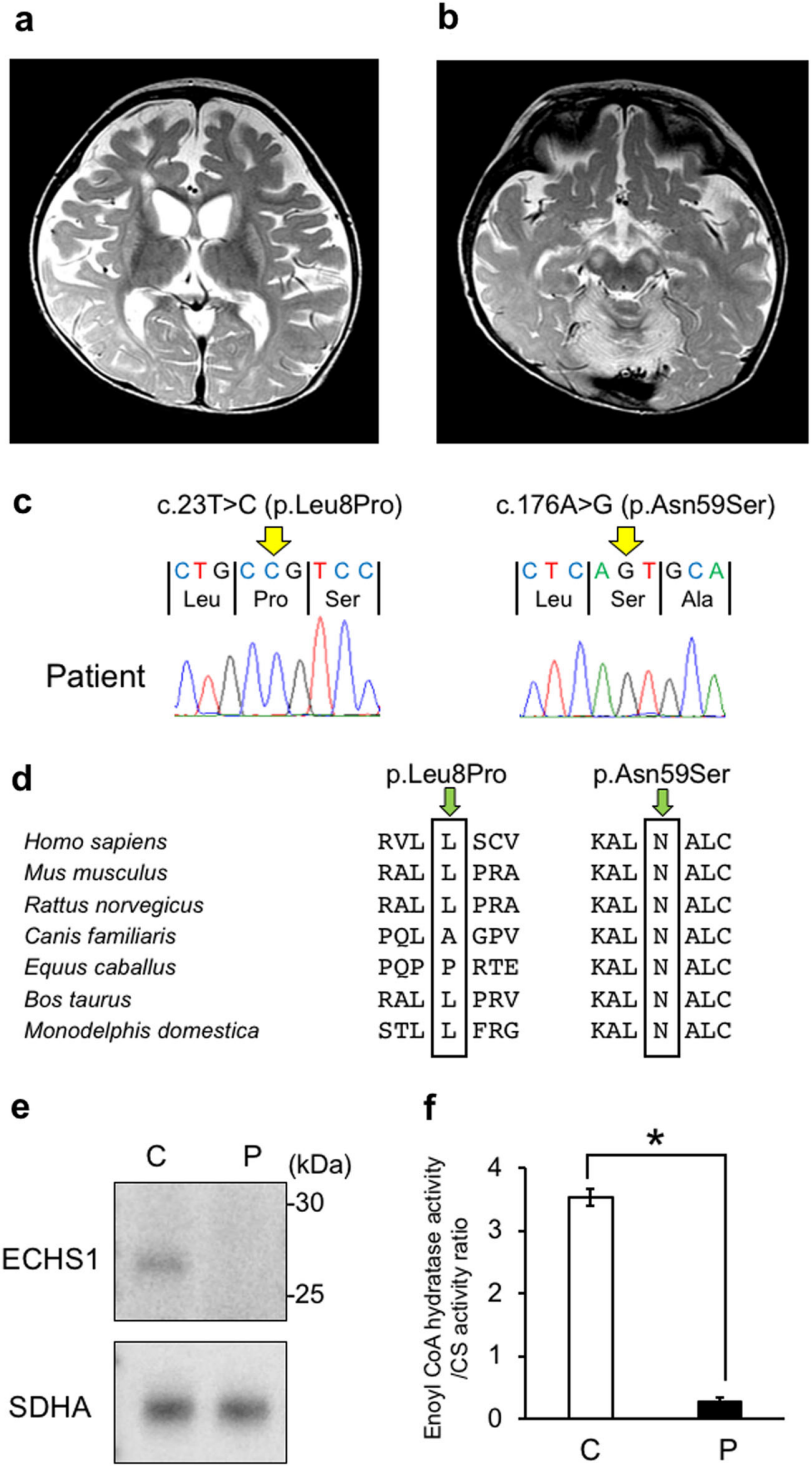
MRI findings and genetic analysis. **a**, **b** T2-weighted brain magnetic resonance imaging at 1 year of age showing bilateral hyperintensity of the caudate nucleus, putamen, globus pallidus (**a**) and substantia nigra (**b**). **c** A compound heterozygous mutation in *ECHS1* in the patient. cDNA clones synthesized from the patient’s mRNA were sequenced. **d** The amino acid sequences of ECHS1 protein in various vertebrates. The leucine at position 8 and the asparagine at position 59 are highly conserved across species. **e** Immunoblotting analysis revealed that ECHS1 protein is not detected in the mitochondrial fraction of the patient’s myoblasts. **f** Enzyme activity of enoyl-CoA hydratase in the mitochondrial fraction of myoblasts is markedly decreased in the patient. Activity levels are expressed as ratios with respect to citrate synthase activity. Error bars indicate standard deviations. *P < 0.01, Student’s *t*-test. C, control; P, patient

To diagnose this patient at the molecular level, we first screened for pathogenic variants in mitochondrial DNA with a MiSeq sequencer (Illumina, USA), but no pathogenic variant was found. We then conducted exome sequencing to identify variants in the nuclear genes. Genomic DNA was extracted from the patient’s blood by standard methods. The DNA was processed with SureSelect XT Human All Exon V6 (Agilent Technologies, Santa Clara, CA, USA). Captured DNA was sequenced using a HiSeq 4000 (Illumina, San Diego, CA, USA) with 150 bp paired-end reads. The reads were mapped to the human reference genome (GRCh37/hg19) by Burrows–Wheeler Aligner (BWA) 0.7.5a-r405. Duplicate reads were removed with Picard 1.99. Variants were identified with the Genome Analysis Toolkit (GATK) v3.2 based on the GATK Best Practice Workflow and annotated with ANNOVAR (2017Jul16).

Two variants of *ECHS1*, namely, NM_004092.4:c.23T>C (p.Leu8Pro) and NM_004092.4:c.176A>G (p.Asn59Ser), were identified and confirmed by Sanger sequencing. As no samples from the patient’s parents were available, we synthesized *ECHS1* cDNA from mRNA extracted from the patient’s muscle biopsy sample and then cloned and sequenced it. The patient’s haplotype phase was confirmed (Fig. 1c).

c.176A>G (p.Asn59Ser) is a known variant associated with LS (HGMD, Professional 2018.3). c.23 T>C (p.Leu8Pro) has also been deposited in dbSNP (Build 150) as number rs775833766, but no association has been reported between this variant and LS. Hence, this is the first report identifying c.23T>C (p.Leu8Pro) as a novel variant associated with LS.

c.23T>C (p.Leu8Pro) was also not described in any other databases, including the ESP6500, 1000 Genomes, Exome Aggregation Consortium, Human Genetic Variation, and ClinVar databases (as of the beginning of May 2018). SIFT and PolyPhen-2 predicted the variant to be “damaging” with a score of 0.02 and “probably damaging” with a score of 0.97, respectively. In addition, the mutated leucine was conserved in mouse, rat, cow, and opossum (Fig. 1d).

To confirm the pathogenicity of these variants, we performed SDS-PAGE and immunoblotting with antibodies against ECHS1 (Sigma-Aldrich; SAB2100643) using the mitochondrial fraction isolated from patient-derived myoblasts. Anti-SDHA antibodies (Invitrogen; 459200) were used as a loading control for mitochondria. The ECHS1 protein was undetectable in the patient, whereas the expression level of SDHA was normal (Fig. 1e). Next, we measured the enzyme activity of enoyl-CoA hydratase in the mitochondrial fraction from patient myoblasts as previously described^4^. Each sample was measured in triplicate and normalized to citrate synthase activity. Enoyl-CoA hydratase activity was markedly decreased in the patient (to 8% of the level observed in controls; Fig. 1f).

Here, we report a patient with a compound heterozygous variant of *ECHS1*. Almost all reported cases of ECHS1 deficiency show LS, with or without cardiomyopathy^5^. This patient’s clinical course was also compatible with typical LS.

This patient had a compound heterozygous variant of *ECHS1*, consisting of c.23T>C (p.Leu8Pro) and c.176A>G (p.Asn59Ser). c.23T>C (p.Leu8Pro) affected mitochondrial transit peptide (amino acids 1–27) in exon 1 and was therefore predicted to impair protein transport into mitochondria. c.176A>G (p.Asn59Ser) in exon 2 has been reported in six cases of ECHS1 deficiency, all of them Japanese^5–7^, which implies that this variant may be common in the Japanese population. Moreover, the variant was located in the catalytic domain (amino acids 30–289). In fact, our functional analysis revealed that both the amount of ECHS1 protein and its level of enzymatic activity were substantially decreased in the mitochondria of the patient’s myoblasts, supporting the pathogenicity of these variants. Because ECHS1 protein was undetectable in mitochondria, we suspected that the patient’s pathology was a consequence of impaired protein transport and/or degradation of mutant proteins.

As is often the case with LS and other mitochondrial diseases, treatment was ineffective, and the patient’s symptoms were progressive. In ECHS1 deficiency, however, it is speculated that some dietary treatments, such as valine restriction, may decrease the production of toxic metabolites and mitigate disease^5^. For diseases that might be treatable, it is important to diagnose them as early as possible by genetic screening, including whole exome sequencing.

## Data Availability

The relevant data from this Data Report are hosted at the Human Genome Variation Database at 10.6084/m9.figshare.hgv.2558 and 10.6084/m9.figshare.hgv.2561.
